# A Narrative Synthesis Review of Out-of-Pocket Payments for Health Services Under Insurance Regimes: A Policy Implementation Gap Hindering Universal Health Coverage in Sub-Saharan Africa

**DOI:** 10.34172/ijhpm.2021.38

**Published:** 2021-05-01

**Authors:** Abigail Nyarko Codjoe Derkyi-Kwarteng, Irene Akua Agyepong, Nana Enyimayew, Lucy Gilson

**Affiliations:** ^1^Faculty of Public Health, Ghana College of Physicians and Surgeons, Accra, Ghana.; ^2^Ghana Health Service, Accra, Ghana.; ^3^Research and Development Division, Ghana Health Service, Accra, Ghana.; ^4^School of Public Health and Family Medicine, University of Cape Town, Cape Town, South Africa.; ^5^Department of Global Health and Development, London School of Hygiene and Tropical Medicine, London, UK.

**Keywords:** Policy Implementation Gap, Universal Health Coverage, Out-of-Pocket Payments, Health Insurance, Sub-Saharan Africa, Low- and Middle-Income Countries

## Abstract

**Background:** "Achieve universal health coverage (UHC), including financial risk protection, access to quality essential healthcare services and access to safe, effective, quality and affordable essential medicines and vaccines for all" is the Sustainable Development Goal (SDG) 3.8 target. Although most high-income countries have achieved or are very close to this target, low- and middle-income countries (LMICs) especially those in sub-Saharan Africa (SSA) are still struggling with its achievement. One of the observed challenges in SSA is that even where services are supposed to be "free" at point-of-use because they are covered by a health insurance scheme, out-of-pocket fees are sometimes being made by clients. This represents a policy implementation gap. This study sought to synthesise the known evidence from the published literature on the ‘what’ and ‘why’ of this policy implementation gap in SSA.

**Methods:** The study drew on Lipsky’s street level bureaucracy (SLB) theory, the concept of practical norms, and Taryn Vian’s framework of corruption in the health sector to explore this policy implementation gap through a narrative synthesis review. The data from selected literature were extracted and synthesized iteratively using a thematic content analysis approach.

**Results:** Insured clients paid out-of-pocket for a wide range of services covered by insurance policies. They made formal and informal cash and in-kind payments. The reasons for the payments were complex and multifactorial, potentially explained in many but not all instances, by coping strategies of street level bureaucrats to conflicting health sector policy objectives and resource constraints. In other instances, these payments appeared to be related to structural violence and the ‘corruption complex’ governed by practical norms.

**Conclusion:** A continued top-down approach to health financing reforms and UHC policy is likely to face implementation gaps. It is important to explore bottom-up approaches – recognizing issues related to coping behaviour and practical norms in the face of unrealistic, conflicting policy dictates.

## Background

Sustainable Development Goal (SDG) 3 has as one of its key targets, “Achieve universal health coverage (UHC), including financial risk protection, access to quality essential health-care services and access to safe, effective, quality, affordable essential medicines and vaccines for all.” At the heart of this concept are the values of social justice and health as a human right. To achieve universal primary healthcare coverage, countries require systems with values that “put people at the centre of healthcare”^1^ and ensure that all people can have the health services they need without suffering catastrophic payments at point-of-service use. Countries must mobilise sufficient funds, decrease out-of-pocket payments for health services, and improve equity and efficiency.^2^ Most high-income countries have attained a reasonable semblance of UHC. All the high-income countries that have managed to do this have relied largely on public financing arrangements rather than private-for-profit health insurance. Evidence from Organisation for Economic Co-operation and Development (OECD) countries shows that private-for-profit insurance while increasing health system resources and consumer choice has equity challenges and tends to raise healthcare expenditure.^3^ The UHC goal remains a challenge for most low- and middle-income countries (LMICs).^4,5^ Only a few LMICs such as Thailand^6^ and Costa Rica have attained UHC, relying on public financing rather than private-for-profit insurance.^7,8^

Globally, the UHC financing arrangements in most countries involve some form of pre-payment from general taxes, insurance premiums or a mix. We use the term “Health Insurance” in this paper to refer to health financing protection from out-of-pocket payments at point-of-service use arrangements that involve both pre-payment and risk pooling regardless of whether the source of funding is general taxes, insurance premiums or a mix.^9^ The OECD proposals for a taxonomy of health insurance arrangements use four main criteria namely, whether: the sources of scheme financing are public or private, the scheme is mandatory or compulsory, participation is on group or individual basis and how the premium is calculated. Public as well as private health insurance schemes can be voluntary or mandatory. Public health insurance schemes are mainly financed through general taxation, mandatory payroll levies and contributions to social security schemes or some combination of the three. The terminology of public health insurance schemes generally overlaps in meaning with the terminology of social health insurance schemes.^10^ Private-for-profit health insurance schemes are predominantly financed through private premiums paid by individuals or groups. A diversity of insurance arrangements exists under the classification of private insurance. Sometimes the boundaries between public and private insurance are blurred with some schemes classified as private-not-for-profit being to large extent social health insurance type schemes in their objectives and financing arrangements.^11,12^ Community health insurance schemes are a form of largely grassroots based health insurance arrangements that often use a combination of public and private-not-for-profit insurance arrangements; that have emerged in LMICs.^13^ Our focus in this review is on all types of health insurance schemes in sub-Saharan Africa (SSA), whether public, private, community health insurance or a combination.

By definition, an out-of-pocket payment for health is a fee paid directly by clients to healthcare providers at the point-of-service use.^14^ Commonly in the literature they are classified as formal and informal. This refers to whether payments are clearly receipted and accounted for as part of the legally authorized systems of fee payment in the health system (formal) or not (informal). In this paper, we expand this terminology because it does not adequately cover all our observations and analysis.

Specifically, we use the terminology of ‘central-policy-intended’ and ‘central-policy-unintended’ as well as ‘peripheral-policy-intended’ and ‘peripheral-policy-unintended’ to qualify the terminology of formal and informal. ‘Central-policy-intended’ refers to payments that are formally sanctioned as part of central-level policy and/or accompanying legislation, administrative rules and procedures in the health system. Central-policy-unintended payments are not sanctioned by the national level policy in its statements, accompanying legislation, administrative rules and procedures.

We use this terminology to distinguish central-level-policy from street level or frontline worker and manager policy. Strictly speaking street level or frontline worker and manager user fee policies not sanctioned by the centre are ‘informal’ payments. However, we found such payments were sometimes formalized, documented in local ‘policy’ statements, receipted in some way or the other and ‘formally’ managed at the periphery. They represented coping behaviour rather than corruption. At the same time, we found that central-policy-intended payments could still be converted into informal payments that are more or less explained by corruption. For example, the situation where health workers collect central-policy-intended fees into their pockets for personal gain and fail to issue receipts. This can happen with poor daytime supervision or during the night when due to poor human resource strength, health facilities cannot provide finance staff for night duties to collect revenue as experienced in some LMIC health facilities. Payments are not receipted accordingly into notional revenue books.

Out-of-pocket payments at point-of-service use especially when large, relative to incomes; are undesirable from an equity and right to health perspective. They potentially lead to catastrophic health expenditure (CHE) especially among the poorest and most vulnerable. Xu et al^15^ analysed household health expenditure in over 50 countries and documented that prepayments and risk pooling for national health system financing through social insurance or general taxes decreases out-of-pocket health payments and prevents CHE. On the other hand, a review of studies in LMICs^16-41^ shows that out-of-pocket payments at point-of-service use, irrespective of the context (eg, absence of health insurance,^16,17,25,26,37,40,41^ community-based health insurance,^23^ private-for-profit health insurance,^24^ national health insurance,^35,36,38,39^ maternal health,^18,21^ mental health,^20^ child health,^19,21,33^ surgical care,^34^ or non-communicable disease^22,29,30^) decrease access to health services and increase CHE for households.

Most OECD countries provide some form of near UHC whether through health insurance, taxation or a mix for almost all their citizens.^42^ In these countries, out-of-pocket payments are central-policy-intended and often take the form of co-payment, deductibles, co-insurance and payment for services not covered by the health insurance.^43^ They are generally designed to form relatively small percentages of total health expenditure.^44,45^ In LMICs, central-policy-intended out-of-pocket payments also exist. They may sometimes form relatively large percentages of the total health expenditure, ranging from under 10% in Malawi to over 90% in China.^46^ These central-policy-intended out-of-pocket payments cover health services and medicines.^46^ They may be a major financing mechanism rather than part of an insurance system. An example is the central-policy-intended co-payment in Rwanda’s health insurance system by relatively high-income groups for non-primary services not covered by health insurance. The poorest are exempt from these payments.^47^

In Kyrgyzstan, Murphy et al^48^ reported that out-of-pocket payments, as co-pays for medicines not covered by the Additional Drug Package of the 1996 mandatory health insurance reform, resulted in poor adherence to antihypertensive medication and caused CHE for the poor. A 2013 study by Barasa et al in Kenya documented the negative effects of direct and indirect out-of-pocket payments for healthcare.^16^ Studies in India, Taiwan and China^49-51^ have shown that out-of-pocket payments cause CHE, especially for the poor.

“Central-policy-unintended payments” have been described as “unofficial,” “informal,” “illegal,” “rent,” “out-of-pocket payments” and “corruption.” It is a topic that is sometimes discussed gingerly because it has complex implicit and explicit power strings and interconnections, which if addressed cursorily, could lead to a cascade of unintended consequences for the health system. In recent times, there is the growing call to more overtly talk about corruption in the health system towards equitably achieving UHC.^52-54^

Some studies have sought to understand central-policy-unintended out-of-pocket payments in the health systems of Iran,^55^ Bangladesh,^56^ Nigeria,^57^ Myanmar^18^ and India^58^ under rent-seeking and informal payments. Other studies cursorily imply the existence of central-policy-unintended payments in health systems of Uganda,^34^ Ukraine,^59^ Ghana,^60,61^ and in 36 LMICs reviewed for acceptability, accessibility, availability and quality of maternal, reproductive and child health services^62^ without focusing on an in-depth exploration. Central-policy-unintended out-of-pocket payments create policy implementation gaps which are discrepancies between policy as ‘intent’ and policy as ‘practice.’^63,64^ In a complex adaptive health system, policy as intent is not always policy as practised due to complex interactions between the actors, processes, content and the context of the policy.^65,66^ The gap between intended and experienced policy is further influenced by the power of frontline workers in their translation of policy into practise as described by Lehman and Gilson in South Africa.^67^

The rationale of this study is to understand this policy implementation gap hindering the attainment of UHC in LMICs of SSA, conscientize health system actors regarding the inequity created by this ignored implementation gap and call policy actors to action.

This study sought to synthesize the published evidence available on the ‘what’ and ‘why’ of central-policy-unintended out-of-pocket payments for services covered by health insurance schemes in SSA. Specifically, we asked: (1) What central-policy-unintended out-of-pocket payments are occurring for services under health insurance schemes? (2) Why are insured clients paying for services covered by the schemes?

In this paper, we briefly examine the theoretical concepts underpinning the study. We describe the methods used for this narrative synthesis review and synthesise our findings into a narrative based on broad themes from our starting theoretical concepts and inductive themes. We end by discussing the findings of this work.

### Theoretical Concepts

The study drew on Lipsky’s street level bureaucracy (SLB) theory, the concept of practical norms, and Taryn Vian’s framework of corruption in the health system for conceptualization and analysis.

Street level bureaucrats are frontline workers of public sector agencies who have autonomy and use discretion in the award of sanctions or benefits to the public. They are effectively policy decision-makers whose actions and inactions become the face of policy as practised. Their actions and inactions can lead to policy as practised different from policy as stated for several reasons. Firstly, the nature of their work is such that it cannot be automated, discretion is unavoidable, and they have to interact extensively with the general public. Secondly, is their condition of work. They are sometimes faced with difficult choices because they do not have adequate amounts of the needed resources to perform. They may face ambiguous and contradictory expectations and demands from the higher authorities in their agencies. Street level bureaucrats thus have to develop some coping mechanisms to handle all these conflicts. These coping mechanisms may unavoidably and unintentionally affect the expression of the policy they are supposed to implement. These coping mechanisms are highly influenced by their independence in a bureaucratic establishment. The actions stemming from their independence are affected by their rationalisation, attitudes and the extensive discretionary power they wield. Policy becomes a bottom-up process.^68^

Vian^52^ in his review paper to describe how rationalisation, opportunities and pressures enable corruption in the health sector, proposed a “framework of corruption in the health sector” that solidifies some concepts and models of corruption propounded by previous scholars through an “organisational view.” He defined corruption as that which “occurs when public officials who have been given the authority to carry out goals which further the public good, instead use their position and power to benefit themselves and others close to them.”

From the perspective of the government official, corruption is stimulated by three main drivers: (1) The official’s ability to justify his actions or rationalise his behaviour based on normative culture, individual attitudes and personality, (2) pressure from clients or financial constraints, and (3) Opportunity to abuse power. The opportunity to abuse power is influenced by monopoly, citizen voice, transparency, accountability, enforcement and discretion.^52^

In his study of corruption in West African states, de Sardan proposes that the gap between official rules and actual practice of the state and state agents is governed by informal rules that generate “practical norms.” This gives an underlying meaning and some structure and system to what one might observe as mere random corruption on the surface. Practical norms are embedded in the routine practices of civil servants through ‘the generalized exchange of favours,’ ‘clientelism,’ ‘culture of impunity,’ ‘arenas of suspicion,’ ‘privilegism’ and contempt for anonymous users, among other norms.^69,70^

## Methods

### Study Site

Almost all countries in SSA have a history of colonisation that influences their current bureaucracies. Their health systems have evolved through post-colonial eras of “free healthcare” for all, structural adjustment, payment of user fees, user fees exemptions and currently the introduction of health insurance towards the attainment of UHC.

The region is characterised by great cultural diversity across and within countries, and high poverty rates despite a wealth of natural resources. More than half of the world’s poor can be found in SSA.^71^ The region has a medium Human Development Index of 0.547 and has 43.2% of the world’s multidimensionally poor. ^72^ Table 1 gives a general context of the countries in which the reviewed studies were conducted.

**Table 1:** Context of the Countries in Which the Reviewed Studies Were Conducted

**Indicator**	**Country**					
	**Burkina Faso**	**Cote d’Ivoire**	**Ghana**	**Kenya**	**South Africa**	**Tanzania**
Total population in thousands (2016)	18646	23696	28207	48462	56015	55572
UHC index of essential service coverage	40	47	47	55	69	43
Life expectancy at birth/years	61.6	57.8	64.1	66.7	64.1	65.5
Population in multidimensional poverty, headcount/%	83.8	46.1	30.1	38.7	6.3	55.4
Human development index	0.452	0.538	0.611	0.601	0.709	0.529
Gender inequality index	0.594	0.638	0.538	0.518	0.406	0.556
Skilled labour force (% of labour force)	5.0	25.5	28.5	40.5	52.2	5.0
Out-of-pocket expenditure as % of total expenditure on health (2014)	39.09	50.81	26.84	26.11	6.49	23.21
Total expenditure on health as % of GDP (2014)	5.0	5.7	3.6	5.7	8.8	5.6
Maternal mortality rate (per 100000 live births, 2017)	320	617	308	342	119	524

Abbreviations: GDP, gross domestic product; UHC, universal health coverage. Sources: The Global Health Observatory (World Health Organization)^73^ and 2020 Human Development Report (United Nations Development Programme).^74^

### Study Design

This study employed a narrative synthesis methodology because it is an appropriate approach when heterogenous literature types involving both qualitative and quantitative work are to be studied^75,76^ and is a good fit for handling diverse groups of studies,^77^ particularly when one seeks to synthesise qualitative evidence.^78^ ‘Textual narrative synthesis’ helped to draw new comprehensive conclusions, from empirical evidence through the use of text rather than data.^76,77,79^

### Information Sources

Literature search was limited to English language literature identified from the International Development Research Centre (IDRC) online data base which provided access to an electronic collection catalogue. This made it possible to search the content of external databases simultaneously with EBSCO databases, using a single interface. EBSCO Databases searched includedAcademic Search Complete, Business Source Premier, CAB Abstracts, EconLit, LISTA MEDLINE, SocINDEX. Other databases, platforms and indexes that were searched via this platform includedCairn.info, Directory of Open Access Journals (DOAJ), ERIC, HeinOnline, JSTOROECD, iLibrary, Persée, SciELO, World Bank eLibrary, eBooks Collection, AGRICOLA, African Journals Online (AJOL), Eldis, Google Scholar, PubMed Central and SciDev.net.

### Search Terms

An initial search in October 2018 for Ghana only, was later expanded to cover SSA. The initial search terms in October 2018 were: “Ghana National Health Insurance” AND “out-of-pocket payment” AND “informal payment” OR “user fees” and “Ghana” AND “National Health Insurance.” The expanded search to include countries in SSA ended on January 31, 2020. It included the search terms: “Africa AND informal fee* AND health insurance,” “Africa AND informal payment* AND health insurance,” “Africa AND informal charge* AND health insurance,” “Africa AND user fee* AND health insurance,” “Africa AND user charge* AND health insurance,” “Africa AND unofficial payment* OR “unofficial fee* OR “unofficial Charge* AND health insurance,” “Africa AND out-of-pocket AND health insurance,” “co-pay* AND Africa,” “Africa AND out-of-pocket* AND Insurance” and “Africa AND health insurance.”

### Inclusion Criteria

The search yielded publications dated 1873-2019. The included articles were English language, peer-reviewed journal articles, covering empirical studies in SSA that documented ‘central-policy-unintended’ out-of-pocket payments under health insurance schemes in the findings or results section.

### Exclusion Criteria

Studies conducted outside SSA, grey literature, reviews and studies that did not address central-policy-unintended payments were excluded.

### Identified Materials

The initial literature search yielded, 1663 publications out of which 666 were peer-reviewed academic journal articles. After removing duplications, reviewing the titles and skimming the abstracts, 19 articles were identified for full text screening. Nine articles were selected based on the inclusion criteria. Review of relevant references yielded one additional article. The final expanded search to include all of SSA identified eight additional articles, making a total of eighteen articles. Figure summarizes the literature search and selection.

**Figure:**
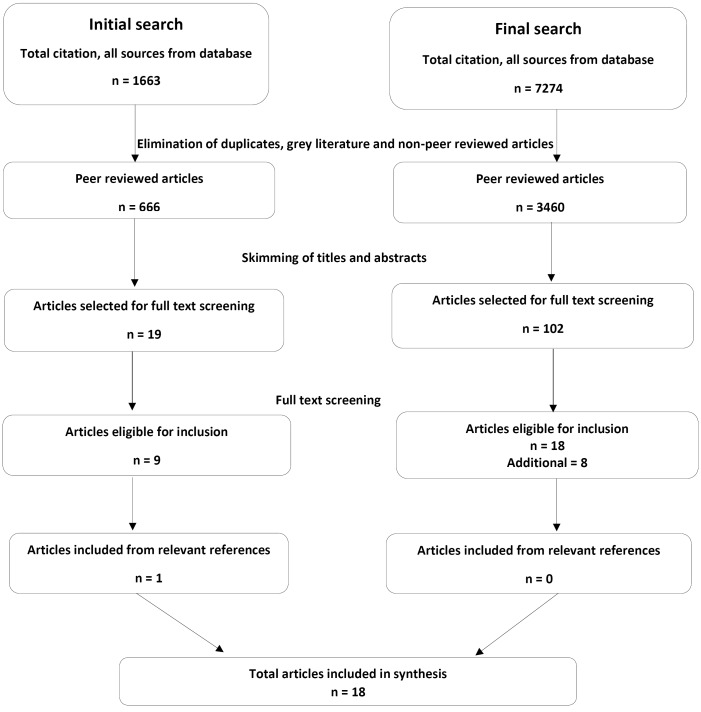
Flowchart of Article Selection Process.

The included articles (Table 2) comprised five mixed methods studies,^62,80-83^ five qualitative studies,^60,84-87^ seven cross-sectional surveys^88-94^ and one quasi-experimental study,^95^ spanning the period 2011-2019.

**Table 2:** Summary of Selected Articles for the Review Process

**Author(s), Title and Year of Publication**	**Country of Study**	**Aim (s) of Study**	**Study Population**	**Methodology (Study Design and Analysis)**	**Framework, Concept, or Theory**	**Type of Health Insurance Scheme**
Agyepong et al, 2016 The "Universal" in UHC and Ghana's National Health Insurance Scheme: policy and implementation challenges and dilemmas of a lower middle-income country	Ghana	Explores NHIS enrolment barriers and facilitators. Provides insights and lessons for attainment of UHC	Adult groups of currently enrolled, previously enrolled, currently uninsured, never insured	Exploratory case study design. Mixed methods. Purposive sampling. Descriptive statistics and thematic analysis	SLB theory. Framework of results developed. Preliminary theory used	Social health insurance (NHIS)
Aidam et al, 2016 The effect of health insurance on out-of-pocket payments, catastrophic expenditures and healthcare utilization in Ghana: case of Ga South Municipality	Ghana	Determines the impact of NHIS membership on healthcare utilization and CHE	Households	Cross-sectional survey.Purposive sampling	Statistical concepts/models used. Hypothesis testing	Social health insurance (NHIS)
Aryeetey et al, 2016 Can health insurance protect against out-of-pocket catastrophic expenditures and also support poverty reduction? Evidence from Ghana’s National Health Insurance Scheme	Ghana	Determines the effect of out-of-pocket expenditure on poverty and the effect of health insurance on out-of-pocket expenditure, catastrophic expenditure and poverty	Households	Cross-sectional survey with follow up survey in 2 years. Three stage sampling procedure	Methodology developed by O'Donnell et al. Pen's parade. Random effects model. Probit model. Durbin-Wu-Hausman endogeneity test	Social health insurance (NHIS)
Ashigbie et al, 2016 Challenges of medicines management in the public and private sector under Ghana's National Health Insurance Scheme - A qualitative study	Ghana	Explores public and private care providers' perception of the challenges and consequences associated with the NHIS' medicines management policies and practices in Ghana	Private, public and mission pharmaceutical providers	Qualitative study. Purposive sampling. Thematic analysis	Faden et al’s concept on Insurance System Medicines Strategies,^96^ Ghana’s Medicines management policy	Social health insurance (NHIS)
Attia-Konan et al, 2019 Distribution of out-of-Pocket expenditures in a sub-Saharan African country: evidence from the national survey of household standard of living, Cote d'Ivoire	Cote d’Ivoire	Estimates the direct payments made to take care of health expenditures and their distribution according to different areas of residence	Households	Cross-sectional survey. Stratified random sampling	Anderson's conceptual framework on factors determining healthcare demand	Voluntary social health insurance (private health insurance)
Beogo et al, 2016 Out-of-pocket expenditure and its determinants in the context of private healthcare sector expansion in SSA urban cities: evidence from household survey in Ouagadougou, Burkina Faso	Burkina Faso	Investigates: (*a*) the level of out-of-pocket expenditure on healthcare, (*b*) the distribution of out-of-pocket expenditure based on its primary components and on the ownership of healthcare facilities, and (*c*) the proximate determinants of out-of-pocket expenditure	Households	Cross-sectional survey. Two-staged cluster random sampling	None	Voluntary social health insurance (private health insurance)
Dalaba et al, 2014 Does the national health insurance scheme in Ghana reduce household cost of treating malaria in the Kassena-Nankana districts?	Ghana	Assesses the impact of NHIS in decreasing malaria treatment cost for households in Kassena-Nankana	Households	Cross-sectional household survey. Random sampling. Principal component analysis technique	None	Social health insurance (NHIS)
Dalinjong et al, 2017 The operations of the free maternal care policy and out of pocket payments during childbirth in rural Northern Ghana	Ghana	Evaluates out of pocket payment and the impact on women during childbirth under the free maternal care policy of the NHIS in poor rural Northern Ghana	Women who gave birth in Health facilities	Convergent parallel mixed methods approach. Purposive sampling. Thematic approach	None	Social health insurance (NHIS)
Dalinjong et al,^a^ 2018 The implementation of the free maternal health policy in rural Northern Ghana: synthesised results and lessons learnt	Ghana	Explores the facilitators, barriers to health access under the free maternal health policy of the NHIS and the implications	Women who had given birth in health facilities	Convergent parallel mixed methods approach. Purposive sampling. Thematic analysis	Synthesis of study results into a framework	Social health insurance (NHIS)
Dalinjong et al, 2018 Has the free maternal health policy eliminated out of pocket payments for maternal health services? Views of women, health providers and insurance managers in Northern Ghana	Ghana	Explores perceptions of cost, actual payment and source of funds for services under the NHIS's free maternal care policy	Women who used maternal health services in health facilities	Convergent parallel mixed methods approach. Purposive sampling. Thematic analysis	None	Social health insurance (NHIS)
Kabia et al, 2019 "We are called the et cetera": experience of the poor with health financing reforms that target them in Kenya	Kenya	To examine the experiences of the poor with health financing reforms and challenges they encounter in accessing their benefits under these reforms in Kenya	Poor people and people in the lowest quintile	Qualitative cross-sectional study. Purposive sampling	conceptual framework adapted from Jacob et al	Social Health Insurance. [Health Insurance Subsidy Program under National Health Insurance Fund]
Kusi et al, 2015 Does the National Health Insurance Scheme provide financial protection to households in Ghana?	Ghana	Determines the effect of NHIS on out-of-pocket health expenditure and CHE for households through a cross-sectional survey in three districts across Ghana	Households	Cross-sectional household survey. Systematic sampling	Hypothesis testing. Methodology by O'Donnell et al	Social health insurance (NHIS)
Macha et al, 2012 Factors influencing the burden of healthcare benefits in Ghana, Tanzania and South Africa	Ghana, Tanzania, South Africa	Explores factors that influence distribution of healthcare benefits focusing on regressive financing mechanisms and the reasons for pro-rich distribution of benefits in terms of affordability, acceptability and accessibility in Ghana, Tanzania and South Africa	Scheme members	Mixed methods approach. Purposive sampling and 2-staged stratified random sampling	Access framework	Voluntary social health insurance (private health insurance), compulsory social health insurance (National Health Insurance Fund, NHIS, community health fund, Tiba Kwa Kadi)
Mpanza et al, 2019 Reasons why insured consumers co-pay for medicines at retail pharmacies in Pretoria, South Africa	South Africa	Explores views about co-payments and factors that influence Pretoria medical scheme members’ co-payments when purchasing prescription medicines at pharmacies, despite insurance status	Scheme members	Exploratory qualitative study. Purposive sampling	None	Voluntary social insurance (private health insurance)
Nguyen et al, 2011 The financial protection effect of Ghana National Health Insurance Scheme: evidence from a study in two rural districts	Ghana	Evaluates the impact of NHIS on out-of-pocket expenditure by households and CHE	Households	Cross-sectional household survey. Two-stage cluster random sampling	Wagstaff and van Door-slater concept of CHE	Social health insurance (NHIS)
Siita et al, 2019 Does capitation affect patient satisfaction and prevalence of out-of-pocket payments in the insured? A propensity score analysis of Ghana's demographic and health survey data	Ghana	Examines the effects of capitation on perceived health service quality and prevalence of out-of-pocket health payments using Ghana's capitation pilot as a case study	Scheme members	Quasi-experimental study. Stratified random sampling. Principal component analysis for robustness Rosenbaum - bounds sensitivity analysis	Principal component analysis, Rosenbaum - bounds sensitivity analysis	Social health insurance (NHIS)
Suchman, 2018 Accrediting private providers with National Health Insurance to better serve low-income populations in Kenya and Ghana: a qualitative study	Ghana and Kenya	Studies the effect of participation in a SHI scheme on private providers’ ability to serve poorer patient populations with quality health services	Poor people	Qualitative study. Purposive sampling	None	Social health insurance (NHIS, National Health Insurance Fund)
Witter et al, 2013 An exploratory study of the policy process and early implementation of the free NHIS coverage for pregnant women in Ghana	Ghana	Explores the policy development process and early implementation of the free NHIS policy for pregnant women in Ghana	Pregnant women and newborns	Qualitative study. Purposive sampling	Thematic framework	Social health insurance (NHIS)

Abbreviations: NHIS, National Health Insurance Scheme; UHC, universal health coverage; SLB, street level bureaucracy; CHE, catastrophic health expenditure; SHI, social health insurance. ^a^Used to differentiate the 2018 papers by same author.

### Quality Criteria

The quality of the articles selected was evaluated based on (1) the use of theory, concepts or frameworks, (2) triangulation, (3) proper documentation of the study process and systematic presentation of data, (4) clear contextualisation of the research, (5) the quality of the empirical evidence presented in the paper, (6) discussion of limitations, (7) ethical considerations and (8) reflexivity.^97-99^ Additional criteria for quantitative studies were, (9) the use of appropriate sampling technique and (10) sample size calculation. Each criterion carried a maximum mark of 1. The papers were assessed based on scores as: Excellent (10), very good (above 7); good (6-7); fairly good (5); poor (below 5). All the included studies scored 6 and above (Supplementary file 1).

### Data Analysis

The principal researcher, a Fellowship student, carried out the literature search, article selection and data extraction process with guidance and supervision from the other researchers who were her fellowship dissertation supervisors. The theoretical concepts, inclusion, exclusion and quality criteria were discussed and agreed upon by all authors before the research process. The second author cross-validated the data extraction and analysis process.

A data extraction sheet (see Box 1) was used to extract data from the eighteen articles individually. An Excel spreadsheet was then developed to organise the extracted data into a matrix to help analyse the data within and across papers. The extracted data was deductively and inductively analysed into sub-themes and themes drawing on the theoretical concepts of corruption, practical norms and SLB taking note of new, emerging themes from the data. Data was extracted from findings; raw data, summarized text, cited text, context and/or arguments in an iterative way.^100^

**Box 1. Data Extraction Template For Included Articles**

Title

Author

Year

Country

Study site

Aim of the study

Type of health insurance

Population under study

What out-of-pocket payments are occurring?

- Timing: Initial/post
- Receipted/not receipted/unknown
- Mode of payment: Cash/kind/favour/gift
- Facility type
- Facility-based/outside the facility
- Type of service: (eg, medicine, laboratory, ultrasound)
- Cadre of health worker involved
- Initiator of the payment: Health worker/patient

Why are the out-of-pocket payments occurring?

(factors influencing the out-of-pocket payment)

- Personal
- Organisational
- Policy factors
- Socio-cultural factors
- Other contextual factors
- Other

Quality Criteria

- (see Supplementary file 1 for details)

Comments

The synthesis entailed identification of constructs, findings, relationships between findings and setting limits for the synthesis.^75,101^

A preliminary presentation of the protocol and initial findings was made at the Faculty of Public Health pre-conference workshop of the Ghana College of Physicians and Surgeons during the December 2018 Annual General Scientific Meeting for peer-review before finalizing the protocol and approach.

## Results

The studies included in the synthesis covered six different countries in SSA with majority from Ghana (Table 2). The findings are presented below organized by the two research questions.

### What Central-Policy-Unintended Payments Are Occurring

We classified the types of services that insured clients made central-policy-unintended out-of-pocket payments for into: medicines,^60,62,88–93,80–87^ diagnostic tests,^62,80,81,85,88-93^ in-patient services,^60,62,80,84,89,90,92,94^ out-patient services,^60,62,88-90,94,95^ blood transfusion services,^62,80^ antenatal,^62,80,88^ labour and delivery services^62,81,83,87,88^ and others (Table 3). Other services included surgeries,^88^ supplies for deliveries^62,80,81,87,89^ and non-specified services.^82,83,85,88^

**Table 3:** Summary of “What” Services Insurance Clients Paid for Out-of-Pocket

**Reference**	**Medicines**	**In-Patient Services**	**Out-Patient Services**	**Blood Transfusion**	**Diagnostic Tests**	**Labour And Delivery**	**Antenatal Service**	**Other**
Agyepong et al, 2016	√					√		√
Aidam et al, 2016	√				√			
Aryeetey et al, 2016		√	√					
Ashigbie et al, 2016	√	√						
Attia-Konan et al, 2019	√	√			√			
Beogo et al, 2016	√				√			
Dalaba et al, 2014	√	√	√		√			
Dalinjong et al, 2017	√	√		√	√	√	√	√
Dalinjong et al,^a^ 2018	√				√	√		√
Dalinjong et al, 2018	√	√	√	√	√	√	√	√
Kabia et al, 2019	√				√			√
Kusi et al, 2015	√	√	√		√			
Macha et al, 2012	√							√
Mpanza et al, 2019	√							
Nguyen et al, 2011	√		√		√	√	√	√
Siita et al, 2019			√					
Suchman, 2018	√	√	√					√
Witter et al, 2013	√					√		√
Total number of articles with type of service	**16**	**8**	**7**	**2**	**10**	**6**	**3**	**9**

^a^Used to differentiate the 2018 papers by same author.

We classified central-policy-unintended payments into ‘peripheral-policy-intended’ and ‘peripheral-policy-unintended.’ The papers were not always explicit and peripheral-policy-intended payments were inferred from instances where payments appeared to be facility-based^62,84,85^ or from phrases such as “double-billing,” “balance-billing,” “top-ups,” “user fees” “*referred to the cashier*” and “co-pays.”^82,83,85-87^ Payments made upon referral were considered peripheral-policy-intended payments since there was no evidence to the contrary. Most referrals were for medicines and insured clients paid more for prescribed medicines than the non-insured, particularly under private insurance schemes.^86,88,92,93^ There was no evidence suggesting that these referrals were a form of rent seeking. Seven of the papers mentioned peripheral-policy-intended payments in the facility and ten of the papers mentioned peripheral-policy-intended payments on referral.

Peripheral-policy-unintended payments were made in cash (7 studies) and kind (5 studies). These payments were described in the papers by words such as ‘informal,’ ‘unofficial,’ ‘unauthorised,’ ‘demands,’ ‘bribes’ and “*outright under the table charges and extortion*.”^60,83-85,88,91,94^ Peripheral-policy-unintended payments in kind were mainly made by pregnant women. They comprised soap, disinfectants and other supplies (rubber mackintosh, sanitary pad) used during labour and delivery.^62,80,81,83,87^ Eleven papers were not specific as to whether the payments were in cash or in kind. The types of peripheral-policy-unintended payments made that could be clearly classified under corrupt practices governed by practical norms^102^ were described as levies (6 papers), gratuity, commission, unwarranted fees and misappropriation (4 papers).

In more than half of the studies reviewed, information was inadequate and some cash payments could not be conclusively categorised as ‘peripheral-policy-intended’ or ‘peripheral-policy-unintended.’^60,62,95,80–82,87,89,90,92,93^

‘Timing’ of the payments (eg, ‘before,’ ‘during’ or ‘after’ service provision) was not significant in determining the type of payment. All payments in private facilities, generally appeared to be higher than in public facilities.^60,85^ Apart from midwives^60,83,87^ and pharmacy or dispensary staff,^82,86^ no other cadre of health workers was specifically mentioned as initiators or recipients of payments in any of the selected articles. Generally, the central-policy-unintended payments appeared to be potentially regressive with clients in low socioeconomic groups most affected.^62,82,86^

### Why Are the Central-Policy-Unintended Payments Occurring

Our analysis suggests that the reasons for central-policy-unintended payments can be explained by our starting theories of SLB, Vian’s theory of corruption, Olivier de Sardan’s concept of practical norms, scheme design and context.

### Context and Design

The countries involved in this review were at various stages of health system financing reforms under the name of health insurance, but with variations in approach (Table 2). In South Africa, Burkina Faso and Cote d’Ivoire health insurance was voluntary private health insurance, mainly for high income earners sometimes managed by for-profit companies. Tanzania and Kenya had a more social health insurance type scheme in the form of their compulsory National Health Fund for formal sector employees. Tanzania additionally had voluntary social health insurance in rural areas through the community health fund and in urban areas, through the *Tiba Kwa Kadi.*Ghana’s National Health Insurance Scheme (NHIS) was universal in intent but had challenges with enforcing compulsory enrolment for the informal sector given the out-of-pocket contributory premiums required additionally to the general tax funding.^83^ Kenya was working on scaling up its National Health Fund to include the informal sector.^60,83,85^ The insurance schemes in Ghana, Tanzania and Kenya had exemptions and waivers for vulnerable groups.^62,82,83,85^ Generally, the poor and lower-income groups in the informal sectors had limited health insurance coverage due to the barrier posed by out-of-pocket premiums and opportunity costs in some cases.^82,83^ All the countries were anticipating the introduction or scale up of a compulsory social national health insurance type scheme towards the attainment of UHC. The health systems and policy issues leading to central-policy-unintended payments identified in the review papers are depicted in Table 4.

**Table 4:** Elements of SLB and Health Policy and Systems Issues Identified in Articles

	**Conditions of work**		**Patterns of practice**		**Others**
	**Inadequate resources relative to tasks**		**Modify concept of work**		**Consequences of alienation**
	Agyepong et al (2016)		Macha et al (2012)		Agyepong et al (2016)
	Ashigbie et al (2016)		Suchman (2018)		Ashigbie et al (2016)
**Source**	Dalinjong et al (2017)				Dalinjong et al (2018)
	Dalinjong et al^a^ (2018)		**Modify concept of clients**		Kabia et al (2019)
	Dalinjong et al (2018)		Agyepong et al (2016)		Macha et al (2012)
	Kabia et al (2019)		Ashigbie et al (2016)		Suchman (2018)
	Kusi et al (2015)		Dalinjong et al (2017)		Witter et al (2013)
	Macha et al (2012)		Siita et al (2019)		
	Nguyen et al (2011)				**Advocacy**
	Witter et al (2013)		**Ration services**		Ashigbie et al (2016)
			Agyepong et al (2016)		Dalinjong et al (2018)
	**Increase demand to meet supply**		Ashigbie et al (2016)		Kabia et al (2019)
	Agyepong et al (2016)		Dalinjong et al (2018)		Suchman (2018)
	Kabia et al (2019)		Siita et al (2019)		Witter et al (2013)
	Macha et al (2012)		Suchman (2018)		
	Nguyen et al (2011)		Witter et al (2013)		**Worker bias**
	Suchman (2018)				Agyepong et al (2016)
	Witter et al (2013)		**‘Rubber stamping’**		Dalinjong et al (2017)
			Ashigbie et al (2016)		
	**Vague/ambiguous/conflicting goal expectations**				
	Agyepong et al (2016)		**‘Creaming’**		
	Macha et al (2012)		Agyepong et al (2016)		
	Suchman (2018)		Kabia et al (2019)		
			Suchman (2018)		
**Health system issues**	**Description and source**	**Health policy issues**	**Description and source**
**Health financing**	**Lack of funds at National level**	**Policy process**	**Hasty process with poor stakeholder engagement**
	Dalinjong et al (2017)	Witter et al (2013)		Witter et al (2013)	
	Suchman (2018)				
				**Poor dissemination for implementation**
	**Delayed reimbursement to providers**		Witter et al (2013)	
	Agyepong et al (2016)	Mpanza et al (2019)			
	Ashigbie et al (2016)	Suchman (2018)		**Poor communication to clients**
	Dalinjong et al (2017)	Witter et al (2013)		Macha et al (2012)	Mpanza et al (2019)
	Kabia et al (2019)				
				**Cumbersome claims process**
	**Low reimbursement rate**		Ashigbie et al (2016)	Witter et al (2013)
	Agyepong et al (2016)	Suchman (2018)			
	Ashigbie et al (2016)	Witter et al (2013)			
				**Poor regulation**
**Lack of resources**	**Human resources, medicines, supplies, equipment**		Agyepong et al (2016)	Mpanza et al (2019)
	Agyepong et al (2016)	Kabia et al (2019)		Ashigbie et al (2016)	Suchman (2018)
	Ashigbie et al (2016)	Kusi et al (2015)		Beogo et al (2016)	Witter et al (2013)
	Dalinjong et al (2017)	Macha et al (2012)		Kabia et al (2019)	
	Dalinjong et al^a^ (2018)	Suchman (2018)			
	Dalinjong et al (2018)	Witter et al (2013)	**Policy content**	**Misunderstanding**
				Agyepong et al (2016)	Macha et al (2012)
**Health system software**	**Mistrust between health system actors**		Dalinjong et al (2017)	Suchman (2018)
	Agyepong et al (2016)	Macha et al (2012)		Dalinjong et al (2018)	Witter et al (2013)
	Ashigbie et al (2016)	Mpanza et al (2019)		Kabia et al (2019)	
	Dalinjong et al (2018)	Suchman (2018)			
	Kabia et al (2019)	Witter et al (2013)		**Complex/conflicting/ambiguous**
				Agyepong et al (2016)	Mpanza et al (2019)
	**Clients’ trust in providers**			
	Kabia et al (2019)	Mpanza et al (2019)	**Policy context**	**lack of insurance accredited health facilities**
				Agyepong et al (2016)	Dalinjong et al (2018)
	**Clients’ lack of trust in providers**		Aidam et al (2016)	Kabia et al (2019)
	Agyepong et al (2016)	Mpanza et al (2019)		Ashigbie et al (2016)	Macha et al (2012)
	Macha et al (2012)	Suchman (2018)		Dalinjong et al^a^ (2018)	Witter et al (2013)

Abbreviation: SLB, street level bureaucracy ^a^Used to differentiate the 2018 papers by same author.

The purely private insurance scheme members mainly used private health facilities to deliver benefits while the other types of health insurance schemes used mainly public health facilities but also some accredited private health facilities. All public health facilities (including quasi-government) were accredited by default while private-for-profit facilities had to undergo a complex accreditation process.^60^ Accreditation for private pharmacies on the other hand was perceived to be easy and efficient. Contrary to public providers, private providers could easily renounce their accreditation status on account of delayed reimbursement, unfair adjustments in claims or low reimbursement rates.^84^ Health facilities were allocated reimbursement restrictions for services based on their level of accreditation such as, a clinic, maternity home, or a hospital, which sometimes limited the service they could provide to insured clients. Facilities were not reimbursed if they provided services above their accredited level of care.^60,83,84,87^ Health insurance reforms were not usually accompanied by the provision of infrastructure and other resources to meet the demand for service. Health insurance clients had to contend with poor road networks,^83^ and challenges in obtaining transportation to accredited facilities or facilities where they could get all needed services. In this regard, the poor and residents in rural areas had more limited access to healthcare than urban dwellers.^62,81,82,84,85,87,91^

Economically, there was generally a lack of funds for the schemes to operate from the macro-level to the micro-level of the health systems in Ghana, Tanzania and Kenya. In South Africa this was a problem between the schemes at the meso-level and the micro-facility level causing clients to co-pay for medicines.^86^ This however did not come out strongly under private health insurance in the two francophone countries. The lack of funds led to delays in reimbursement to health facilities lasting between 3-9 months,^60,83-85,87^ non-review of service tariffs by insurance schemes and low reimbursement rates. Health facilities became less credit worthy and had to deal with suppliers who refused to participate in tenders, leading to high operating costs.^84^ Insurance officials in Ghana also complained of lack of funds at district offices.^83^

Policy processes were sometimes not carefully thought through, documented and disseminated for implementation. In Ghana, the promise of foreign aid, the millennium development goals and interest of powerful national level actors affected the hasty agenda-setting, formulation and implementation of NHIS’ “free” maternal care policy. Major stakeholders felt they had not been informed of policy details and that the policy content was not well-disseminated. Key stakeholders involved in the policy process were pacesetters in creating implementation gaps by charging central-policy-unintended out-of-pocket fees. There was no prior financial planning or budgeting and actors were not sure of the sustainability of “free” services with no clear documented sources of revenue.^87^ Providers and insurance officials generally considered the claims processing and vetting activities to be quite laborious and cumbersome since most facilities did not have electronic health information management systems.^83,87^ Policy regulation processes were either absent or poorly implemented.^60,83-87,93^

There was a general sense of mistrust between various actors in the health system because actors were generally not transparent and accountable to each other. Private insurance scheme members and regulators thought that schemes took advantage of members’ “reliance-on-scheme” and preferred to sponsor other interests rather than invest in members’ benefits.^86^ Clients in Tanzania also distrusted the management of the community health fund.^82^ The insurance clients and health workers in Ghana felt they had been deceived by government and that health insurance did not serve the purpose for which it was introduced.^62,83,84^ Insurance beneficiaries in Kenya felt “deceived” and “tricked” by the government when they had to make out-of-pocket payments in health facilities.^85^ In Ghana, Tanzania and Kenya people would not enrol or use health insurance even when enrolled because they still paid for services covered by the scheme.^60,82,83,85^ Insurance schemes did not trust the claims submitted by service providers. They suspected that the claims were inflated,^60,83,86^ fraudulent^60,84,87^ or that providers were double-billing the clients and the scheme.^60,87^ They denied claims and adjusted payments with minimal explanations apparently based on these suspicions.^84^ Clients and providers in Ghana distrusted insurance officials because they sometimes collected informal charges from clients and private providers to speed up registration and accreditation processes.^60,83,84^

Clients generally trusted they got expensive but better quality and more responsive care from private than public facilities. Clients trusted the care they received based on the expertise of the attending provider. Thus health workers in lower-level public facilities were perceived to be inexperienced and unskilled.^60,82,83,85^ Some clients on the other hand trusted the health workers in the public health facilities because they were more knowledgeable and skilled.^85^ Due to the fear of misdiagnosis, and the apparent financial freedom offered by health insurance, clients usually “shopped” with the same complaint.^84,87^ Providers in Ghana perceived this as a moral hazard and suspected clients gave their medication to uninsured relatives or sold them for personal gain.^84^ The higher level public facilities were also preferred because they offered a wider range of services.^60^ Doctors and pharmacists used their power to influence clients to co-pay for expensive medicines in South Africa based on trust in their expertise.^86^ Similarly, private insurance clients in Cote d’Ivoire and Burkina Faso who consulted with doctors made more out-of-pocket payments than those not seen by doctors and the non-insured.^92,93^

### Street Level Bureaucracy

The elements of SLB, extracted using the 2010 edition of Lipsky’s work^103^ are illustrated in Table 4.

### Discretion

Frontline worker discretion was an important explanatory factor for central-policy-unintended out-of-pocket payments. Health workers used their discretion to determine who got ‘free’ service and who paid for service. When facilities in Ghana suspended ‘free’ health services to insurance clients, insured pregnant women were ‘rubber stamped’^103^ (approved) to receive free services due to strong political and global influence towards the attainment of millennium development goals. They still had to pay for the very expensive services to sustain the system.^84^ Some pregnant women had to pay more than other clients because they were farmers, married, uneducated, nulliparous^80^ or belonged to a different religion.^83^ This is because health workers perceived these categories as high-risk clients who made more demands on the already constrained health system or as groups that complied easily with the central-policy-unintended payments needed to maintain a functional health system. An unintended consequence of health worker differentiation between clients (worker bias).^103^ On the other hand, clients perceived to cause less strain on resources and workload were also exempted from making central-policy-unintended out-of-pocket payments by ‘creaming.’^103^

The autonomous use of discretionary power appeared to be influenced by rationalisation and justification based on moral/ ethical lines, societal norms, informal rules, pressures from clients, individual values or the prevailing sanctions. The use of discretion created variation in implementation of policy. Vulnerable groups such as pregnant women and orphans were sometimes exempt from making out-of-pocket payments (advocacy)^103^ and in other cases had to pay like everyone else based on discretion. A poor rural woman who lived with her brother’s orphaned children always received all services for free while others were treated with contempt and had to make payments under insurance.^85^ Health workers sometimes personally paid the peripheral-policy-intended bills of needy insured clients.^60^ Some health managers stocked medicines for insurance clients while others stocked medicines not on insurance lists for profit.^84^

### Conditions of Work

Macro and meso-level health system and policy issues, created conditions of work that forced frontline workers to adopt ‘coping’ patterns of practice (Table 4). This generated peripheral-policy-unintended and peripheral-policy-intended fees at a cost to clients. Agency goals and expectations were sometimes conflicting and ambiguous, such as, health workers being expected to provide “free” services despite resource constraints and reimbursement uncertainties.^60,82,83^ They faced daily dilemmas such as; ‘do I treat an emergency case for free and not get reimbursed, charge the client for the service, refer to a distant referral centre knowing the client would default or could die on the way, or make clients buy the unavailable supplies for the procedure?’

Apart from private facilities attending to private insurance clients, all other facilities had to grapple with chronically inadequate resources mainly due to lack of funds at facility level from delayed or low insurance reimbursements and poor resource allocation. There was a lack of human resource, equipment, drug and non-drug consumables across all facility types. It was difficult to maintain or procure equipment, upgrade facilities, motivate and recruit staff.^62,80-85,87-89^

Facilities could not replenish their stocks due to inadequate funds, fall-out with suppliers or because the limited funds were being prioritised for other activities.^60,62,81-85,87-89^ These resource constraints were sometimes not so clearly evident in the papers that centred solely on private insurance.^86,92,93^

Health insurance reforms generally led to increased demand for health services. The increase in utilization resulted in increased workload and waiting times in health facilities, especially in public than private health facilities, across South Africa, Ghana, Tanzania and Kenya.^60,82,83,85,87,88^

Health workers modified the concept of their work and their clients through the creation of routines and simplifications to cope with conditions of work, conserve resources and prepare for unpredictable emergencies. Though this helped frontline workers to deal with the pressures of work, it also resulted in unintended “opportunity costs” for clients from lateness, absenteeism, strikes, disrespect and poor responsiveness (consequences of health worker alienation^103^).^60,62,82-85,87^ Work was modified in some cases to shorter opening hours in Tanzania, Ghana and South Africa, apparently to make time for additional administrative work.^82^ Long queues^85^ and the provision of very little information helped control clients’ behaviour.^60^ Health workers also sometimes rationed services to insurance clients by imposing indirect costs (waiting),^103^ denying services or referring^103^ them to other facilities where they sometimes had to pay out-of-pocket.^60,62,83-85,87,95^

### Ambiguous Policy Content

The policy content was not always clear to all actors involved in the policy process. It was sometimes froth with misconceptions, ambiguous goal expectations, and no provision for unintended consequences.^83^ Ghanaian actors in health facilities, district and regional levels did not clearly understand the fund disbursement process even though they appreciated the diversity in the sources of funding for the scheme.^83,87^ The language of the policy was sometimes too complex for insurance clients and health workers to understand in South Africa. Clients were sometimes not sure what their benefits entailed.^86^ The concept of capitation was poorly understood by health workers and clients in Ghana and Tanzania.^60,87^ Scheme members in Tanzania also complained of poor ‘benefits-communication’ system.^82^ Waivers and exemptions could not be granted because actors did not understand the criteria for eligibility^82,83^ or did not know that they existed.^82^ Some insured clients in Kenya made out-of-pocket payments because they did not know how to use insurance.^60^ This lack of understanding meant that health workers charged for services which were covered and scheme members were not sure what they had to pay or not pay for.

### Monopoly

Although enrolment with an insurance scheme was supposed to provide financial access to clients, this access was limited. The few facilities available to clients controlled the types of services they received. In some cases, clients still had to make payments for services covered by the scheme, incur opportunity cost from travelling or waiting in long queues, endure contempt from health workers or pay more for responsive services in private facilities. There were instances where poor clients who were referred to other facilities due to faulty equipment or stock-outs defaulted.^85^ The lack of district hospitals sometimes hindered enrolment in rural areas.^83^ There was not much competition between health facilities and the options available to insurance clients always had limitations. Opting out or using their power of choice was not effective as sanctions for improvement in the health system.

### Practical Norms (Olivier de Sardan) and Corruption (Vian’s Theory)

#### Practical Norms

There were instances where health workers abused power for their personal or collective gain rather than as coping behaviour. It was sometimes difficult to separate out practical norms from corruption. The different types of payments identified in this synthesis which sometimes reflected widespread “practical norms” and sometimes isolated corrupt behaviour included levies, commissions, misappropriation, unwarranted fee for service and gratuity. Where these central-policy-unintended payments were influenced by the informal rules of practical norms (Table 5); they overlapped with and were similar to some patterns of practice of street level bureaucrats in some cases making it difficult to clearly separate out the dominant behaviour drivers.

**Table 5:** Elements of Practical Norms and ‘Corruption Complex’ Identified in the Articles

**Reference**	**Elements of Practical Norms**									
	**Systemic Corruption**	**Impunity**	**Contempt for Anonymous Users**	**Formal Versus the Real**	**Each-One-for-Oneself-ism**	**Rooms of Suspicion**	**‘Privilegism’**	**A Double Language**	**Clientelism**	**Dependence on Donor Support**
Agyepong et al (2016)	X	X	X	X				X		
Aidam et al (2016)	X									
Aryeetey et al (2016)	X									
Ashigbie et al (2016)	X	X			X	X	X			
Attia-Konan et al (2019)										
Beogo et al (2016)										
Dalaba et al (2014)										
Dalinjong et al (2017)										
Dalinjong et al^a^ (2018)										
Dalinjong et al (2018)				X						
Kabia et al (2019)	X	X	X	X				X	X	
Kusi et al (2015)										
Macha et al (2012)										
Mpanza et al (2019)			X	X	X	X			X	
Nguyen et al (2011)	X	X		X						
Siita et al (2019)										
Suchman (2018)	X	X				X	X			
Witter et al (2013)	X			X		X	X			X
Total number of articles with the practical norm present	8	5	3	6	2	4	3	2	2	1

^a^Used to differentiate the 2018 papers by same author.

Generally, the elements of practical norms and corruption seemed to run through various levels of the health system and interacted with each other. They were evident in all the countries included in this synthesis. Insured clients lamented about its infectious nature, “…*there is corruption in Kenya, corruption is everywhere*….”^85^

Health insurance staff took advantage of their privileged position as public officials and used their discretionary power to ‘levy’ other actors in the health system. They would sometimes delay reimbursement to private pharmacies that refused to pay “kickbacks.”^60,83-85,88^ These pharmacies in turn used their discretionary power to opt-out of the scheme or refused to serve insurance clients.^84^ Health workers on the other hand also demanded bribes and unofficial fees (levy) from clients. Midwives, who were seen as very essential health workers because of the need to improve maternal health indicators globally, were observed by Witter et al^87^ to have acted with ‘impunity,’ withdrawing services in peripheral facilities when they were cautioned against corrupt practices. They collected drug consumables from facility pharmacies (misappropriation) for personal gain, levied clients^83^ and demanded ‘gratuity’ from mothers who delivered in health facilities at significant cost to clients.^87^ Dispensary staff in Kenya audaciously bargained with clients over the cost of medicines covered by insurance. Although the clients complained and were opposed to these corrupt practices, they too were sometimes partakers, paying ‘commissions’ to skip long queues since there was no other way to get by the ‘system.’^85^

#### Vian’s Theory: Transparency

Actors in most of the countries reviewed did not clearly understand how the various insurance policies worked. Information dissemination and clarity was generally poor. Actors from national level to the frontline did not understand why reimbursements were delayed.^60,85-87^ No explanations were given for delayed reimbursements in the countries affected. In South Africa, for example, neither regulators nor pharmacists could explain why insured clients had to co-pay for medicines. The co-payment arrangements were described as ‘unpredictable,’ ‘inconsistent,’ ‘complicated,’ ‘dynamic’ and ‘not transparent.’ To increase profit, pharmacists failed to explain the cost-benefits of cheaper generic medicines to clients who had to co-pay on account of brand loyalty.^86^ In all the countries, it was as if this confusion was needed to generate placid acceptance of the gap in policy implementation.

#### Vian’s Theory: Enforcement

Health insurance policies generally appeared to be poorly regulated. There was no evidence of sanctions for corruption, and coping strategies were not streamlined.^60,83-87,93^ There was no mechanism in place to monitor the “provider shopping” by clients that put undue strain on health system resources in Ghana.^84^ Private insurance holders were charged indiscriminately for services leading to co-payments in South Africa and bills that were about three times that of the non-insured in Burkina Faso.^86,93^ It appeared the regulators did not have the moral justification to call perpetrators to order since the charging of central-policy-unintended fees sometimes appeared to keep defunct health systems functional.

#### Vian’s Theory: Citizen Voice

There was very little evidence of citizen’s engagement in the policy process. They had limited power to influence policy because they lacked clear knowledge of their insurance benefits, were non-voluntary, were sometimes poor and were afraid of being mistreated. Without appropriate and effective complaint mechanisms, clients were not enthusiastic about complaining.^85^ Even rich private insurance clients were reluctant to ask questions about their payments because they just wanted to get better and were afraid that they would not understand the complex explanations.^86^ Therefore, citizen voice could not ensure answerability and enforcement of sanctions.

## Discussion

Although a few studies have explored central-policy-unintended out-of-pocket payments at point-of-service use from the perspective of coping behaviour and/ or corruption in SSA,^54,104,105^ this study is different in its effort to synthesize the existing literature from the perspective of a policy implementation gap in the context of health insurance reforms. It is an important effort at this time given the context of SDG-3 and the push for UHC, and to leave no one behind.

This study draws on Vian’s framework, SLB theory and the concept of practical norms to help health policy actors understand the policy implementation gap of central-policy-unintended out-of-pocket payments by health insurance clients for insured services in SSA. The use of frontline discretionary power broadly leads to coping or corruption in the expression of this policy implementation gap. Corruption might be for personal gain or as part of a “corruption complex” influenced by practical norms. Powerless health insurance clients lacked expert knowledge on health and the content of their insurance policies. They did not trust that the health system had their interest at heart. They were afraid, voiceless and paid bribes as their expression of power to attain that which is their right (health). Monopoly or competition, citizen voice, transparency, enforcement, and the use of discretion affects the expression of accountability in the health system. Proper accountability at all levels of the health system eventually affects the use of discretionary power in the expression of practical norms or street level bureaucrats’ decisions, actions and inactions in policy implementation.

Context matters, and macro and meso-level contextual factors affect the experience of clients at the frontline of the health system. Accountable leadership and governance at the macro level of country health systems translates into equitable allocation and prioritisation of resources for health systems, formal/informal norms and values within the health system such as trust. These affect the rationalisation and justification of frontline workers’ behaviour.

Despite an increasing push for health insurance – whether social, tax funded, community-based or mixed–type financing reforms in SSA, attention is not being paid to the importance of a holistic understanding of the health systems and contexts for these reforms. Much of the push appears to be from a top-down perspective to policy-making and yet most of the conditions stipulated by Hogwood and Gunn^64^ for effective policy implementation via the top-down approach were not met by the countries in this narrative synthesis review. The bottom-up expression of the policy that is occurring at implementation level appears to be conveniently ignored. Despite their importance for successful interventions in complex system, effective stakeholder engagement and voice, including frontline workers in co-production of the design and implementation of policy, clear policy statements, and fitting of design to context – including resources for implementation do not seem to feature as much in the design considerations as the more technical econometric issues. And yet the literature shows that frontline providers and managers’ discretionary power has effect on policy implementation in LMICs.^106^ Policy ambitions were also bigger than the resources available despite the literature on the coping behaviour that results when what the policy dictates is not possible on the resources provided. Insufficient health financing, invariably resulted in central-policy-unintended payments for sustainability and sometimes for personal gain as described by Gaal et al.^107^ A review on informal payments for maternal health services also revealed that poor health financing by government decreased trust and forced health workers to adopt coping strategies which could be formal or informal.^108^ Health workers were not just behaving badly by charging out-of-pocket payments for personal gain but in some cases were trying to make sense out of their reality by coping.

The lack of transparent leadership and governance as well as accountability as a health system value influenced the implementation of insurance policies and the creation of the policy implementation gap of unintended out-of-pocket payments. Accountability puts limits on the abusive use of discretionary power at all levels of the health system. It informs judicious allocation of resources, transparency in policy processes, fosters trust as a health system value and influences the rationalisation and justification of actors’ actions. Accountability is expressionless without appropriate sanctions and the application of sanctions without appropriate allocation of resources for implementation can result in unintended consequences. Other studies in LMICs also propose the lack of accountability as a cause of informal payments and corruption.^54,109^

The cultural norm of gift giving that influenced the demand of gratuity by midwives in this study was described by other studies proposing that the cost imposed by these gifts on the patient and relatives could make them legal or illegal.^54,110,111^ In the context of this study, this practical norm appeared to be quite expensive to some households and bordered on corruption.

Although there is evidence that other LMICs in SSA are moving towards UHC through health insurance reforms,^112^ coping behaviour, practical norms and corruption explain the out-of-pocket payments not stipulated by the policies that end up limiting access to health^113^ and puts the poor at a disadvantage^114^ despite lofty policy intents to achieve universality and equity.

We recommend that policy-makers adopt an integrated iterative approach to policy formulation, implementation and evaluation with extensive stakeholder involvement at all levels. Policy content should be clearly communicated to ensure actor empowerment and involvement. Reforms should occur in tandem with the scale-up of health resources and infrastructure to meet demand. Moral reflexive leaders^115^ should be tasked to transform health systems.^116^

### Limitations

This study adds to the literature on corruption and policy implementation gaps in LMICs. It drew on three theoretical concepts to explain a complex policy implementation gap because a single theory could not suffice. For example, SLB could not explain out-of-pocket payments under private health insurance. Although we identified overlaps in theory tenets such as the expression of contempt for anonymous users (practical norm) which could also be explained under the consequences of alienation and worker bias in SLB, the theories did not exhaustively explore all the influences on frontline discretionary power leading to policy implementation gaps. Further empirical studies drawing on other theories such as actor interface analysis^117^ or contextual interaction theory^118,119^ could be beneficial.

The inclusion criteria employed for the selection of literature might have excluded some papers of relevance to this review. The selected papers were from six countries in SSA and this might limit the generalisability of this research to other countries. This study raises further questions and sets the stage for further empirical studies looking at the role of leaders and managers in the creation of this policy implementation gap, how much central-policy-unintended out-of-pocket payments are being made by insured clients, the role of trust, power and accountability in propounding this implementation gap, why the reimbursement delays, if the referrals were ‘rents’ or ‘coping,’ and which cadre of health workers are involved in charging central-policy-unintended out-of-pocket payments. The cross-sectional studies gave limited information on the ‘why’ of central-policy-unintended out-of-pocket payments from Francophone countries.^92,93^ In addition to the Francophone studies, only South Africa addressed private social health insurance.^82,86^ This limits the study’s generalisability to private insurance schemes and francophone countries.

## Conclusion

The policy implementation gap of out-of-pocket payments for health services covered by insurance is real in SSA. This implementation gap works against equity and UHC. The poor and vulnerable groups who are supposed to benefit the most from health insurance reforms in terms of financial risk protection and financial access to health are the biggest losers. A continued top-down approach to health financing reform and UHC policy is likely to continue to face implementation gaps. It is important to explore bottom-up approaches – recognizing issues related to accountability, trust, coping behaviour and practical norms in the face of unrealistic and sometimes conflicting policy dictates in relation to resources available and other policies that also have to be implemented; as well as corruption, rather than a simplistic assumption that non-compliance and/or fees unintended by the policy are all corruption.

## Acknowledgements

This study was funded and supported by the Alliance for Health Policy and Systems Research, Health Policy Analysis Fellowship programme under the World Health Organisation. The primary author received invaluable mentoring and peer support from the fellows and mentors of the programme as part of the 2017 cohort.

The IDRC funded Consortium for Mothers, Children, Adolescents and Health Policy Systems and Strengthening project supported this work through sponsorship for health policy and systems research capacity building in West Africa through a pre-doctoral training program in Accra (6 weeks) in 2017 and provision of IDRC library access and its document delivery service for the literature search.

## Ethical issues

This study is the first phase of a fellowship research approved by the Ghana Health Service Ethics Review Committee. Since only published peer reviewed literature was used for this research there was no requirement for ethical clearance.

## Competing interests

Authors declare that they have no competing interests.

## Authors’ contributions

ANCDK carried out the study and was the primary author. IAA, NE and LG supportively supervised the research process. IAA edited the initial manuscript. All authors contributed to the conceptualisation of the study and the final draft.

## Funding

This work was partly funded by the Alliance for Health Policy and Systems Research through the Health Policy Analysis Fellowship Programme, the IDRC funded Consortium for Mothers, Children, Adolescents and Health Policy Systems and Strengthening project, and the Ghana Health Service’s paid study leave award programme.

## Authors’ affiliations

^1^Faculty of Public Health, Ghana College of Physicians and Surgeons, Accra, Ghana. ^2^Ghana Health Service, Accra, Ghana. ^3^Research and Development Division, Ghana Health Service, Accra, Ghana. ^4^School of Public Health and Family Medicine, University of Cape Town, Cape Town, South Africa. ^5^Department of Global Health and Development, London School of Hygiene and Tropical Medicine, London, UK.

## Supplementary files

Supplementary file 1. Quality Criteria for Selection of Papers for the Study.
