# Supplementary material for: A Narrative Synthesis Review of Out-of-Pocket Payments for Health Services Under Insurance Regimes: A Policy Implementation Gap Hindering Universal Health Coverage in Sub-Saharan Africa
Source: Int J Health Policy Manag. 2021 May 1;10(7):443–61. doi: 10.34172/ijhpm.2021.38 (PMC9056140; doi:10.34172/ijhpm.2021.38)
Supplement: Supplementary file 1 — Quality Criteria for Selection of Papers for the Study. [file ijhpm-10-443-s001.pdf]

Article title: A Narrative Synthesis Review of Out-of-Pocket Payments for Health Services Under Insurance Regimes: A Policy Implementation Gap Hindering Universal Health Coverage in Sub-Saharan Africa

Journal name: International Journal of Health Policy and Management (IJHPM)

Authors' information: Abigail Nyarko Codjoe Derkyi-Kwarteng[1,2]*, Irene Akua Agyepong[1,3], Nana Enyimayew[1], Lucy Gilson[4,5]

[1]Faculty of Public Health, Ghana College of Physicians and Surgeons, Accra, Ghana.

[2]Ghana Health Service, Accra, Ghana.

[3]Research and Development Division, Ghana Health Service, Accra, Ghana.

[4]School of Public Health and Family Medicine, University of Cape Town, Cape Town, South Africa.

[5]Department of Global Health and Development, London School of Hygiene and Tropical Medicine, London, UK.

(*Corresponding author: nyarkocodjoe@yahoo.co.uk)

Supplementary file 1: Quality Criteria for Selection of Papers for the Study

Table S1: Quality criteria for selection of papers for the study

Reference	Theory/ Framework/ Concept	Triangulation	Documentation and data presentation	Reflexivity	Contextualisation	Quality of empirical evidence	Limitation	Ethical consideration	Sampling	Sample size calculation	Total
Agyepong *et al* (2016)	1	1	1	0	1	1	0	1	1	N/A	**7.0**
Aidam *et al*. (2016)	1	0	1	0	1	0.5	1	0	1	1	**6.5**
Aryeetey *et al*. (2016)	1	0	1	0	1	1	1	1	1	0	**7**
Ashigbie *et al* (2016)	1	0	1	0	1	1	0	1	1	N/A	**6.0**
Attia-Konan *et al*. (2019)	1	0	1	0	1	1	1	1	1	0	**7.0**
Beogo *et al*. (2016)	0	0	1	0	1	1	0	1	1	1	**6.0**
Dalaba *et al*. (2014)	0	0	1	0	1	1	1	1	1	1	**7.0**
Dalinjong *et al*. (2017)	1	1	1	0	1	1	1	1	1	1	**9**
Dalinjong *et al*.[a] (2018)	0	1	1	0	1	1	1	1	1	1	**8**
Dalinjong *et al*. (2018)	0	1	1	0	1	1	1	1	1	1	**8**

Kabia *et al*. (2019)	1	1	1	0	1	1	1	1	1	N/A	**8**
Kusi *et al*. (2015)	1	0	1	0	1	1	1	1	1	1	**8.0**
Macha *et al*. (2012)	1	1	1	0	1	1	0	1	1	0	**7.0**
Mpanza *et al*. (2019)	0	1	1	0	1	1	1	1	1	N/A	**7.0**
Nguyen *et al*. (2015)	1	0	1	0	1	1	1	1	0	0	**6.0**
Siita *et al*. (2019)	1	0	1	0	1	1	1	1	1	0	**7.0**
Suchman (2018)	0	1	1	0	1	1	1	1	1	N/A	**7.0**
Witter *et al*. (2013)	1	0	1	0	1	1	1	1	1	N/A	**7.0**

[a] Used to differentiate the 2018 papers by same author
